# Enhancing urban traffic management through shared autonomous electric vehicles and dynamic simulation

**DOI:** 10.1371/journal.pone.0311848

**Published:** 2024-12-06

**Authors:** Jingfa Ma, Hu Liu, Lingxiao Chen

**Affiliations:** Shanghai Institute of Technology, School of Railway Transportation, Shanghai, China; University of Shanghai for Science and Technology, CHINA

## Abstract

In the face of rapid urbanization and the increasing number of vehicles, urban centers are struggling with traffic congestion. This study presents a dynamic travel strategy using the MATSim platform to schedule urban travel, incorporating a model for shared autonomous electric vehicles. The model is evaluated using a baseline scenario for Shanghai, exploring the effects of vehicle range, charging capabilities, and power supply strategies on the uptake of shared autonomous electric vehicles. Results indicate that enhancements in vehicle range and charging efficiency slightly decrease the use of autonomous vehicles by 2.5%, as the existing vehicle specifications already meet daily travel needs in Shanghai. Additionally, the transition from traditional charging stations to a battery-swapping system does not significantly alter overall travel behavior of shared autonomous electric vehicles. These findings provide insights into the deployment of intelligent traffic systems to alleviate urban traffic congestion.

## Introduction

Transportation systems are crucial for modern urban life, yet rapid urban population growth has led to increased motor vehicle numbers, exacerbating challenges like traffic congestion [1]. Shanghai, a major economic hub and one of China’s most densely populated cities, exemplifies these issues with severe congestion. According to the "2023 China Urban Transportation Report," Shanghai is now the third most congested city in China, with its congestion index rising to 1.834 from the previous year. This persistent congestion not only disrupts urban mobility but also impedes broader urban development.

To address these challenges, intelligent transportation systems (ITS) are essential. ITS integrate advanced technologies, including sensors, computers, electronics, and sophisticated communication and management strategies, to enhance the safety and efficiency of urban transportation networks [2]. In recent years, research on shared transportation systems has delved deeper into alleviating urban traffic pressure and enhancing transport efficiency. For instance, studies have shown that integrating the location-routing problem of green logistics with eco-friendly packaging can effectively reduce the environmental impact of transportation [3]. The multi-depot vehicle routing problem has been optimized further through smart recycling pricing and transportation resource sharing, improving resource utilization and logistics efficiency [4]. Additionally, research on the truck-drone hybrid routing problem, which considers time-dependent travel times on roads, has proposed new combinations of transportation modes to boost efficiency [5]. The two-tier multi-depot multi-period location-routing problem significantly enhances the overall efficiency of logistics systems by simultaneously handling pick-up and delivery tasks [6].Given its high travel demand and congestion levels, Shanghai is a critical case study for implementing such systems to alleviate traffic issues and support ongoing rapid development.

The rise of mobile internet, smartphones, and innovative automotive technologies has facilitated the emergence of shared autonomous driving. This modern transportation mode provides membership-based access to autonomous vehicles for short durations, managed by third-party organizations. Users incur time-based charges and use digital platforms for reservations, vehicle collection, and payments.

Shared autonomous driving offers various operational models: round-trip, where vehicles are returned to their pickup points; one-way, allowing drop-off at different locations but posing fleet management challenges; and the increasingly popular point-to-point service, enabling departures and arrivals at user-defined locations. Enhanced by advanced data processing technologies, the point-to-point model aims to improve vehicle distribution efficiency across the network.

In the realm of global autonomous driving vehicles, initial research and testing of autonomous driving technology were conducted by Google on traditional fuel-powered vehicles in 2010. Due to the rapid development of electric vehicles, evolving autonomous driving technologies have been increasingly applied to electric vehicles. With advancements in technology, infrastructure, and public policy, from the provision of partial autonomous driving functions in 2014 to the support of full autonomous driving capabilities by 2024, autonomous driving technology in electric vehicles has continuously improved. Consequently, an increasing number of electric vehicles are adopting advanced autonomous driving technologies. The application of autonomous driving technology in point-to-point mode shared electric vehicles has sparked considerable discussion and interest.

Given the nascent stage of shared autonomous vehicles, the intelligent transportation sector is navigating uncharted territory, raising crucial inquiries about vehicle selection, charging methods (whether to use charging stations or battery swaps), and optimal placement of charging stations to enhance user engagement and minimize expenses. The traditional method of trial and error, though prevalent, falls short in efficiently scaling autonomous vehicle services for point-to-point sharing in China. This necessitates a refined model to accurately forecast the dynamics of supply and demand for these vehicles.

The complexity of shared autonomous vehicle systems, encompassing elements like "user, location, parking, vehicle, and energy," presents challenges due to the interconnected feedback between supply and demand. For example, the future vehicle availability at a location could be influenced by various factors including the charging needs and expected arrival times of vehicles en route, which may be affected by traffic congestion. Consequently, the development of a shared autonomous vehicle framework demands a comprehensive understanding of spatial and temporal vehicle data. Traditional mathematical models may not fully capture this complexity, whereas simulation techniques can effectively represent the actions and interactions of distinct agents or events, fulfilling the shared autonomous vehicle model requirements.

In light of this, our study proposes a dynamic travel strategy that uses an adaptive scheduling method to optimize vehicle dispatching and user demand matching during simulation iterations. This is implemented using the MATSim platform, an enhanced open-source multi-agent traffic simulation framework tailored for dynamic travel scenarios, to build a shared autonomous driving model. The aim is to enhance intelligent transportation and alleviate traffic congestion.By integrating travel, road, and public transportation data, we constructed and evaluated a foundational scenario for Shanghai.This study examines how variables such as vehicle range, charging speed, and power options affect user demand.The goal is to lay a scientific foundation for the development and design of autonomous vehicle sharing systems, ultimately promoting efficient urban travel planning and advancing autonomous driving technology to mitigate traffic congestion in Shanghai.

The structure of this paper includes:

1. Introduction: Discussion on research background and real-world needs.
2. Related Review: Review of relevant research achievements, specifying the key focus and significance of this study.
3. Dynamic Travel Planning Optimization: Convergence optimization of MATSim platform for dynamic travel.
4. Autonomous Driving Shared Car Module Constructiont: Detailed description of the development of the shared autonomous driving model, including the design of baseline scenarios and simulated situations.
5. Results: Presentation of model validation and scenario results.

Finally, conclusions and future prospects are discussed.

### Literature review

When facing the challenge of traffic congestion, one of its core issues is the imbalance between supply and demand. The increase in traffic demand directly affects the continuous traffic flow on roads in densely populated areas, which are not only the main arteries for inter-city traffic but also critical points directly affected by congestion within cities. Various factors contribute to traffic congestion, including accidents, adverse weather conditions, slow-moving heavy vehicles, sudden vehicle breakdowns, traffic violations, congestion at intersections, and infrastructure conditions [7]. In response to these challenges, the application of intelligent transportation systems has been proposed, which includes the application of dynamic travel and autonomous driving technologies. The former can reasonably plan traffic behaviors and activities based on travelers’ activity plans, while the latter can avoid traffic congestion by dynamically planning vehicle operations in real-time, thereby improving travel efficiency and alleviating urban congestion. This study has several limitations. Firstly, the simulations rely on data concerning vehicle range, charging speed, and user behavior, which may not fully capture the complexity of real-world scenarios. Secondly, the analysis is constrained by available data, which may not encompass all variables influencing traffic patterns and vehicle usage. Despite these limitations, this study makes a significant contribution to the field of intelligent transportation systems. The study introduces a dynamic travel planning model using MATSim to optimize urban mobility and facilitate the application of shared autonomous electric vehicles. The research provides valuable insights into how vehicle range and charging infrastructure affect the adoption and usage of autonomous driving vehicles. Furthermore, the findings highlight the potential of battery swapping systems as an alternative to traditional charging stations. These contributions lay the groundwork for future research, urban traffic management, and practical applications of autonomous driving technology.

### Application of dynamic travel

[8] Provided system predictions via mobile devices to passengers of stochastic multi-service public transit networks, including transit travel times and arrival times at stops, utilizing a dynamic optimal strategy search approach [9]. Studies have shown that informing drivers in advance about real-time traffic conditions and potential delays is crucial. In situations of traffic congestion, effectively influencing drivers to change their original route selections through dynamic travel information applications using variable message signs is considered an effective approach. A predictive model based on historical traffic data and current traffic information was proposed, presenting a data-driven traffic path planning algorithm aimed at finding the shortest travel time [10]. In recent years, methods for addressing traffic congestion have focused on optimizing path algorithms, controlling traffic signals, and reducing vehicle density to increase average vehicle speeds and thus mitigate the impacts of traffic congestion. Dynamic travel management is applied to single public transit networks and subway networks based on timetable-based road networks, or used in predictive models to find the shortest travel time traffic paths.

Although there have been studies on dynamic travel and traffic management, few have directly applied dynamic travel planning to address traffic congestion with shared autonomous vehicles. This paper utilizes the MATSim platform as a supporting tool, which stands for Multi-Agent Transport Simulation. This open-source software, based on agent-based simulation methods, can simulate the travel behaviors of a large number of individuals within a city or region, including mode choice, path selection, and time distribution, thereby predicting traffic flow, congestion situations, and travel time distribution, among other traffic indicators.

### Support for autonomous driving implementation

In the realm of shared autonomous vehicle simulation studies, researchers primarily deploy two kinds of models: agent-based and event-based. Agent-based models are adept at depicting the behavior and interaction of autonomous entities within a framework. For instance, Heilig and colleagues applied this approach to estimate the demand for shared autonomous vehicles around Stuttgart over a period of one week [11]. Zhang and team adopted it to examine the dynamics within integrated multimodal transport systems, analyzing how vehicle and charging system characteristics interact [12]. Wang and his group explored the benefits of agent-based simulations for enhancing shared mobility services [13]. Meanwhile, Creutz and associates utilized MATSim, Meanwhile, Creutz and his colleagues studied the appeal of shared autonomous vehicles using MATSim (Multi-Agent Traffic Simulation), an agent-based microscopic traffic simulation tool capable of modeling individual behavior in large-scale transportation systems; they gradually refined the simulation framework to include features such as station capacity and advanced reservation mechanisms, and established the base scenario for their experiments in Zurich, to model the appeal of shared autonomous vehicles, progressively refining their simulation framework to include features like station capacity and advanced reservation mechanisms, setting up a foundational scenario in Zurich for their experiments.

This area of study has attracted extensive examination into the dynamics of supply and demand for shared autonomous vehicles, covering aspects such as the variation in membership and fleet sizes, optimization of parking space, adjustments in shared vehicle pricing, vehicle redistribution, and the competitive landscape among providers.

On the contrary, discrete event models map out sequences of individual occurrences over time, mirroring the ongoing shifts within a system’s state [14]. Jefferies and team employed such a model to evaluate electric bus systems, focusing on scheduling and the charging infrastructure [15]. Similarly, Wang and colleagues utilized an event-based approach to model how the demand for shared autonomous vehicles and parking spaces might evolve [16].

Though both model types have been scrutinized, discrete event models stand out for their ability to capture the dynamic nature of events over time, reflecting systemic changes. Yet, few have managed to incorporate specific functionalities crucial for simulating shared autonomous vehicle scenarios, such as:

1. Allowing autonomous vehicles to compete with other transport modes directly, with algorithms that help agents choose more efficient travel options.
2. Adjusting autonomous vehicle travel times based on current traffic conditions.
3. Enabling simulations to dynamically adjust routes and departure times based on real-time data.
4. Providing detailed spatial and temporal output, essential for precise modeling and analysis of transportation networks.

### Advantages of MATSim

Implemented in Java as an open-source simulation platform, MATSim excels at depicting traffic jams, modeling intricate supply-demand dynamics, facilitating agent interactions, and supporting agent learning and adaptation. It delivers outputs with detailed geographical and chronological precision. This suite of features establishes MATSim as the go-to software for analyzing dynamic travel behaviors and the intricacies of shared autonomous vehicle simulations.

1. In MATSim, shared transportation systems are positioned to compete against traditional transport methods, leveraging a sophisticated scoring system to guide agents toward the most efficient travel options.
2. It models the impact of traffic congestion on the duration of shared transportation journeys.
3. MATSim boasts the functionality to adjust travel paths and scheduling in real-time, responding dynamically to changing conditions.
4. It offers outputs that are rich in spatial and temporal details, enhancing the depth and utility of simulations.

## Modeling methods

### Problem statement

With rapid urbanization and an increasing number of vehicles, especially in megacities like Shanghai, traffic congestion is becoming increasingly severe. Congestion not only disrupts residents’ daily travel but also negatively impacts the city’s economic development.Current research primarily focuses on sharing conventional fuel vehicles, while electric autonomous vehicles face additional challenges due to their range and charging time limitations.Efficiently integrating vehicle sharing and autonomous driving technologies to enhance traffic efficiency and reduce congestion is an urgent issue needing resolution.The widespread adoption of electric vehicles requires effective charging infrastructure.However, traditional charging station models may face spatial and temporal limitations in urban areas. The effectiveness and feasibility of battery swapping systems as an alternative solution require further exploration and validation.Addressing these issues, this study aims to develop a dynamic travel planning model to optimize intelligent transportation systems, enhance the efficiency of shared autonomous electric vehicle applications, and ultimately alleviate traffic congestion.

### Optimization of convergence criteria for dynamic travel planning algorithms

MATSim dynamically simulates agents’ travel behavior by following a detailed process that includes initialization, plan generation, iterative simulation, evaluation, and optimization. The objective is to optimize travel paths and activity scheduling, reflecting real-time traffic conditions and agent interactions.

**Fig 1:**
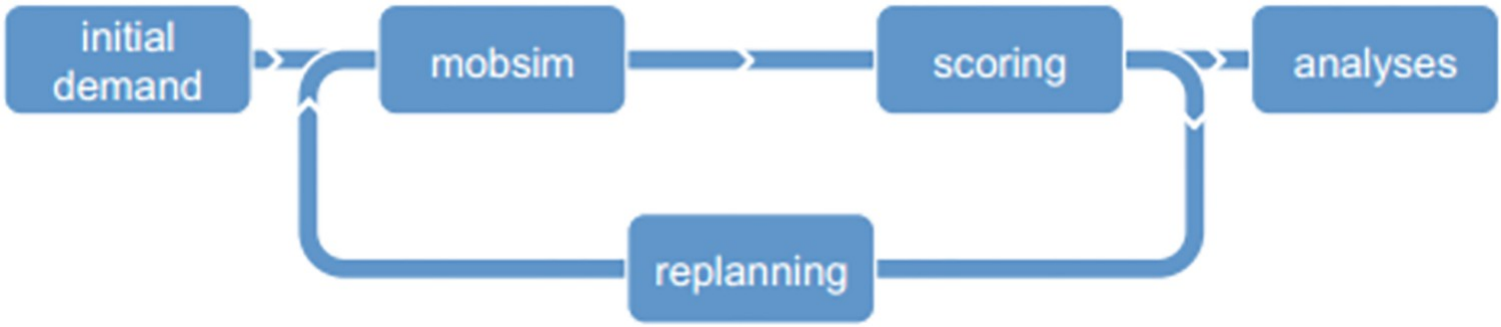
Five main steps of MATSim.

The MATSim Simulation Process is illustrated in Fig 1.

1. Initialization: Input data such as city maps, road networks, agent locations, and travel demands are prepared.
2. Plan Generation: Agents’ travel demands are converted into detailed activity and movement plans.
3. Iterative Simulation: Agents execute their plans in a simulated environment, interacting with other agents and simulating traffic flow over a set period.
4. Evaluation: Agents’ behavior and simulation results are evaluated to score and optimize future plans.
5. Optimization: Plans are adjusted based on evaluation scores to improve travel efficiency and path selection.

This cycle continues until a termination condition is met, such as a preset time or traffic stability, allowing MATSim to assess the impact of various traffic strategies and policies.However, a high number of iterations will also lead to increased model complexity and increased computing time and resource requirements.The questions are as follows:

1. Extended Computation Time: Simulating multiple plans for each agent results in a significant computational workload, especially with numerous travel plans.
2. Increased Model Complexity: As the number of travel plans grows, maintaining and analyzing the model becomes more challenging.
3. Higher Demand for Computational Resources: More travel plans require additional computational resources, which may be limited.

One of the main factors affecting the number of iterations in MATSim is the number of plans generated by the agents. Currently, MATSim offers two plan selection settings:

1. Unlimited Plans: This setting allows for the generation of an unlimited number of travel plans. While it comprehensively covers all possible travel scenarios, it results in excessive computational demands and increased model complexity.
2. Limited to 5 Plans: In this setting, each agent retains only the top 5 highest-scoring plans. This reduces the computational burden by limiting the number of plans, but it may lead to the elimination of potentially optimal plans.

To address the aforementioned issues, this paper proposes a method for dynamically adjusting the number of plans. Initially, a higher limit on the number of plans is set at the beginning of the iterations. As the iterations progress and the system stabilizes, the limit on the number of plans is gradually reduced. This approach maximizes the retention of high-scoring plans and minimizes the risk of deleting globally optimal plans due to a fixed limit.

### Model optimization

Through this improved approach, the main innovation lies in setting convergence conditions. In the computation of the mean, the method involves iterating through each plan in the plan list, summing their scores, and then dividing by the number of plans to obtain the average. Subsequently, in computing the standard deviation, the method calculates the square of the difference between each plan’s score and the mean, sums these squared values, divides by the number of plans, and finally takes the square root to obtain the standard deviation. Next, the percentage change between the current mean and the mean from the previous iteration is calculated and compared against a preset threshold to determine if convergence has been reached. Similarly, the percentage change between the current standard deviation and the standard deviation from the previous iteration is calculated and compared against the threshold. This method assesses convergence by comparing the rate of change between the current mean and the previous iteration’s mean, as well as between the current standard deviation and the previous iteration’s standard deviation.

$$mean=\frac{\sum Score_{i}}{i}$$ (1)

mean is the average score of the current iteration."

i represents the ith iteration (i = 1, 2, 3…)

Score_i_ is the plan score of the ith iteration.

$$std=\sqrt{\frac{\sum {\left(Score_{i}-mean\right)}^{2}}{i}}$$ (2)

std is the standard deviation of the current iteration.

$$\frac{\left|mean-prevMean\right|}{prevMean}<threshold$$ (3)

$$\frac{\left|std-prevMean\right|}{prevStd}<threshold$$ (4)

prevMean is the average score of the previous iteration. prevStd is the standard deviation of the previous iteration. threshold is the predetermined threshold.

In summary, this method provides a simple yet practical approach to assess the convergence status of scores. It involves calculating the percentage change in mean and standard deviation and comparing them with mean and standard predefined thresholds to make judgments on scores. The advantage of this method lies in offering a straightforward yet effective way to determine the convergence status of scores. By computing the percentage changes in mean and standard deviation, it enables a quick assessment of whether the scores are stabilizing, thus allowing for early termination of iterations. This is particularly significant for problems requiring numerous iterative computations, as it can save time and computational resources.


$$S_{\mathrm{plan}}=\sum_{q=0}^{N-1}S_{act,q}+\sum_{q=0}^{N-1}S_{\mathrm{trav},\mathrm{mode}(q)}$$ (5)

In the basic functionality of S_plan_, the final score is determined by calculating the sum of S_act,q_ for all activities and the sum of scores for S_trav, mode (q)_ all trips (or non-trips).

Here, N represents the number of activities. Trip q follows activity q closely. In the scoring process, the last activity is merged with the first activity to generate an equal number of trips and activities.


$$S_{\mathrm{trav},q}=C_{\mathrm{mode}(q)}+\beta_{\mathrm{trav},\mathrm{mode}(q)}\cdot t_{\mathrm{trav},q}+\beta_{m}\cdot \Delta m_{q}+(\beta_{d,mode(q)}+\beta_{m}\cdot \gamma_{d,mode(q)})\cdot d_{\mathrm{trav},q}+\beta_{\mathrm{transfer}}\cdot x_{\mathrm{transfer},q}$$ (6)

C_mode (q)_ is a constant associated with a specific travel mode.

β_trav, mode (q)_ is the direct marginal utility of the travel mode.

t_trav, q_ is the travel time between activity locations q and q + 1.

β_m_ is the marginal utility of money (typically a positive value).

Δm_q_ is the monetary budget change caused by the fare or toll of the ticket or complete journey (typically a negative value or zero).

β_d, mode (q)_ is the marginal utility of distance (typically a negative value or zero).

γ_d, mode (q)_ is the monetary distance rate for a specific travel mode (typically a negative value or zero).

d_trav, q_ is the travel distance between activity locations q and q + 1.

β_transfer_ is the penalty for transferring in public transportation (usually a negative value).

x_transfer, q_ is a binary variable (0/1) used to indicate whether there was a transfer between the previous and current trips.

Since the model used in MATSim integrates multiple factors and diverse decision-making behaviors of individuals, with the optimization of the entire system as its goal, transportation travel is just one aspect of it. The final score is determined by the combined effect of multiple factors, with transportation travel score occupying a relatively small proportion. To make the improvement in score more pronounced, the revised formula is as follows:

$$S_{\mathrm{plan}}=\sum_{q=0}^{N-1}S_{act,q}+{10\sum }_{q=0}^{N-1}S_{\mathrm{trav},\mathrm{mode}(q)}$$ (7)

The revised formula can increase the proportion of transportation travel score in the total score. However, it is important to note the applicability and limitations of this method. The selection of thresholds needs to be adjusted according to specific problems and datasets. Different problems may require different thresholds to determine convergence.

### Support for shared electric autonomous vehicles

Recent research in simulating shared electric autonomous vehicles has concentrated on effective methodologies to replicate this evolving phenomenon. Such simulations must account for the mechanics of electric vehicle (EV) charging cycles and constraints related to battery capacity. Typically, battery limitations are gauged by the vehicle’s State of Charge (SOC), translating into the feasible distance it can cover, like an SOC of 100km indicating a remaining travel distance of 100km before needing a recharge.

Addressing the nuances of EV charging and discharging stands as a pivotal aspect of these models, with many opting for a stringent charging protocol to streamline the process. This requires vehicles to be fully charged or to remain at the charging point until ready for subsequent use. Repoux et al. introduced a minimum charge requirement for vehicle utilization, a premise that might introduce biases, particularly under high demand scenarios, potentially diminishing car-sharing adoption [17]. A more nuanced approach, termed "partial" charging, necessitates only enough SOC for the vehicle’s imminent journey. This approach was analyzed by Ilgen and Hock, who evaluated the impact of various vehicle types on car-sharing operations through real charging process simulations [18]. Moreover, He et al. explored how battery capacity affects EV sharing with partial charging strategies, noting reduced usage with limited battery capacity [19]. Xu and colleagues developed a model to assess battery health and the viability of on-demand partial charging in determining EV fleet sizes [20].

However, these analyses typically rely on historical usage data and do not fully incorporate the influence of external factors like competition with alternative transportation modes or urban traffic congestion on car-sharing demand. This indicates a gap in fully integrating car-sharing into urban transport system simulations, with existing models for electric autonomous car sharing missing these critical considerations.

Despite MATSim’s contributions to addressing certain aspects, it currently falls short in simulating electric car sharing, assuming traditional gasoline vehicle operation without accounting for electric range constraints.

Thus, it’s clear that:

1. There’s a pressing need for a model that realistically simulates electric car sharing within urban settings, considering factors such as the effect of traffic congestion on travel times, competition with other transport forms, and the need for dynamic route adjustments and detailed output data. This study introduces a MATSim-based electric car-sharing model employing a partial charging approach.
2. In MATSim-centered car-sharing research, although round-trip and free-floating data are commonly used for validation, empirical studies on one-way electric autonomous car sharing are sparse. The burgeoning field of one-way electric autonomous car sharing in China offers a fertile ground for validating new simulation models tailored to this modality.

### Shared electric autonomous vehicles module development

The principal distinction between electric vehicles (EVs) and their gasoline-powered counterparts centers on their travel range and refueling or recharging duration. Gasoline vehicles can cover extended distances on a full tank and refuel rapidly, whereas EVs, with evolving battery and charging technologies, generally offer limited ranges and require more time to recharge. An EV’s readiness for use is significantly influenced by its State of Charge (SOC), a vital measure that predicts how far the vehicle can travel before needing a recharge. Additionally, the time needed to recharge to full capacity is determinable through the charger’s specific rate.

In practical simulations that mirror real-world usage of EVs, each virtual vehicle is assigned distinct SOC values, following a normal distribution. This setup also includes mechanisms for evaluating vehicle readiness, managing the charging process, and monitoring energy use. With these parameters, the model assesses each electric vehicle’s energy needs and charging status, thereby ascertaining if the vehicle’s battery holds enough charge for the upcoming journey. This involves intricate simulations of vehicle charging, availability, and energy consumption dynamics, rooted in the vehicle’s SOC and interconnected sub-modules, guiding the evaluation of whether an EV can undertake the next trip as planned.

Eq (8) represents the logic of the electric vehicle charging module. We assume that the charging rate is twice the normal rate when the SOC of the vehicle is below 80% of the total range. Charging will stop when the electric vehicle reaches its maximum battery capacity.


$$\begin{array}{c} SOC_{v,t_{j}}=SOC_{v,t_{i}}+Charged\\ \mathrm{Charged}=\min(CR_{v}\times (t_{j}-t_{i}),MR_{v}-SOC_{v,t_{i}})\\ t_{j}-t_{i}\geq 0 \end{array}$$ (8)

Where $SOC_{v,t_{i}}$ is the State of Charge (SOC) of electric vehicle v at time t_i_, CR_v_ is the charging rate, MR_v_ is the maximum range of the electric vehicle (EV), and Charged represents the charging power between time intervals t_i_, t_j_.

Charging equipment E_i_ is an auxiliary component in the charging module. When an electric vehicle is in the charging state, one charging equipment will be occupied: if E_i_ is occupied, E_i_ = 1; if E_i_ is free, E_i_ = 0.

Eq (9) illustrates the logic of the electric vehicle energy consumption module. We assume that the battery’s energy consumption is linearly proportional to the vehicle’s travel distance, meaning that as the vehicle travels farther, the battery’s energy consumption increases proportionally.


$$\begin{array}{c} SOC_{v,d_{j}}=SOC_{v,d_{i}}-Consumed\\ \mathrm{Consumed}=PC_{v}\times (d_{j}-d_{i}) \end{array}$$ (9)

Where $SOC_{v,d_{i}}$ is the State of Charge (SOC) of the electric vehicle when it has traveled distance d_i_, PC_v_ is the power consumption per unit distance, and represents the consumption power consumed between d_i_ and d_j_ distance intervals.

Eq (10) demonstrates the logic of the electric vehicle availability assessment module. When the state of charge (SOC) of the electric vehicle is insufficient to support the next scheduled trip, the vehicle cannot be picked up.


$$\begin{array}{c} \mathrm{if}\;\frac{SOC_{v,t}}{PC_{v}}-TD_{m,\;t}>0,\;electricvehicleVisavailable\\ \mathrm{else},\mathrm{electricvehicleVisnotavailable} \end{array}$$ (10)

**Fig 2:**
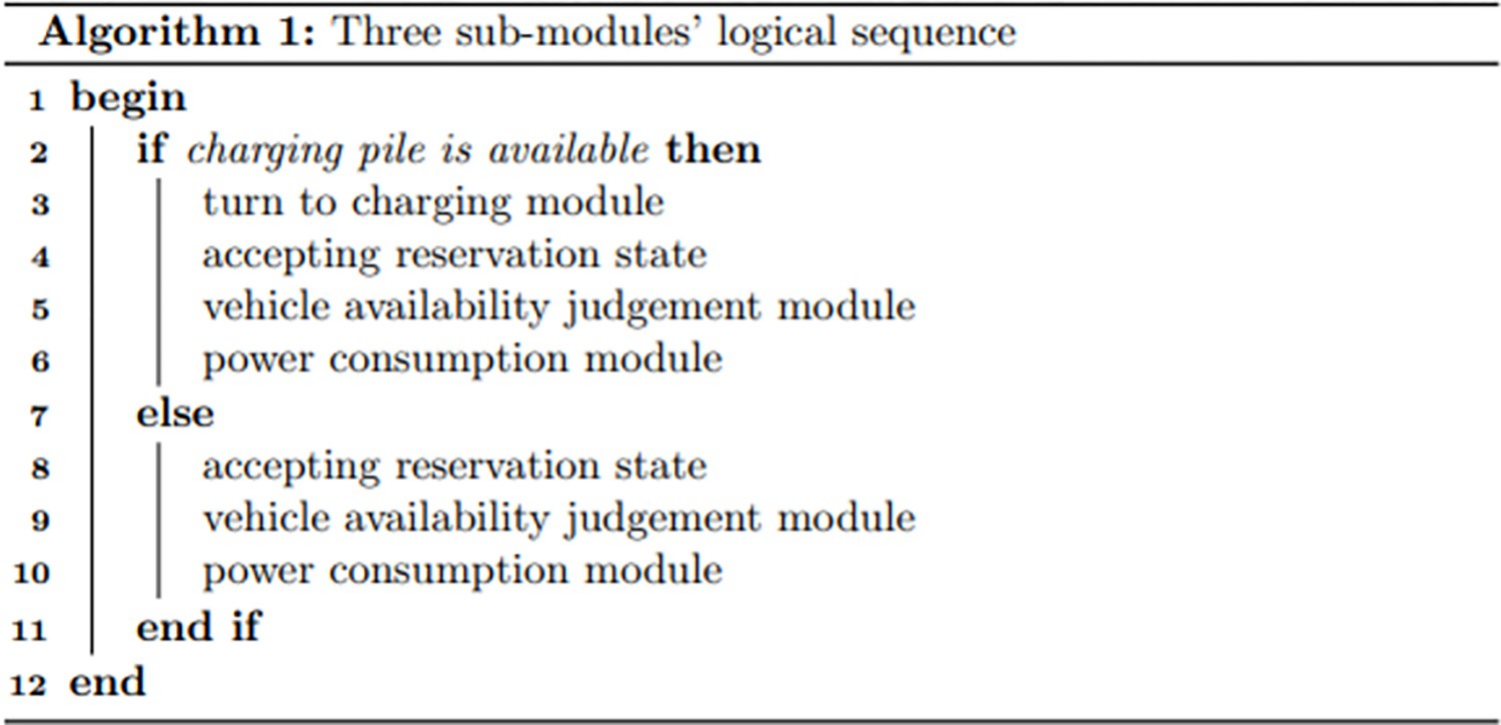
The logical relationships of the three sub-modules.

In this context, SOC_v,t_ is the State of Charge (SOC) of electric vehicle v at time t, PC_v_ denotes the energy consumption per unit distance, and TD_m,t_ signifies the reserved travel distance for a member m of a shared car at time t. The logical relationships of the three sub-modules are presented below in Fig 2.

### Simulation in MATSim

In MATSim, the simulation of mobility covers various modes including cars, public transit, and autonomous vehicles, all interacting within the same environment and subject to traffic congestion.

The newly developed module for electric autonomous vehicles has been successfully incorporated into the MATSim framework. This enhancement leverages MATSim’s capabilities while introducing features for charging and discharging of virtual autonomous shared electric vehicles, complemented by the installation of charging stations.

For users of the autonomous shared electric vehicle model, the process involves evaluating if the chosen vehicle has enough charge for their journey at the time of booking. Upon return, it’s required that the vehicle be fully recharged for the next user. The operation of point-to-point autonomous shared electric vehicles includes several steps:

1. Users find and reserve the closest available vehicle at the station, ensuring it has adequate battery life for their trip, thus making it unavailable for other users during this time.
2. The vehicle is then disengaged from its parking spot or charging unit and moves to a location accessible to the user.
3. Upon trip completion, the vehicle autonomously returns to its station. Should it need recharging, it connects to a charger. After the charging process, it’s parked in a specific spot, ready for the next user.

### Shanghai baseline scenario

For the specified module focusing on point-to-point electric autonomous vehicles, a foundational scenario has been created to both validate the model and examine the interaction between supply and demand for these vehicles. This baseline scenario encompasses elements from both the supply side, including the network and vehicle stations, and the demand side, featuring daily activities and travel patterns, alongside various parameter settings. The city of Shanghai, China, was chosen as the setting for this research, spanning an area of 6,340 square kilometers and home to roughly 24 million people.

#### Basic data support

The simulation’s resource provision encompasses multiple components: (1) a comprehensive multimodal network; (2) schedules and pathways for public transport; and (3) infrastructure for autonomous driving cars including stations, parking, charging amenities, and the vehicles themselves. Network data, sourced from the Shanghai Institute of Traffic Research, provided insights into the city’s transportation network structure, such as topology, optimal speeds, capacity of roads, and the availability across various transport modes. To enhance data precision, adjustments were made like altering lane counts and optimizing speeds to align with real-world conditions observed via Gaode Maps street views, focusing primarily on significant urban routes and highways.

Public transportation data regarding timetables and routes were also obtained from this institute, formatted to be compatible with MATSim. This included detailed information on bus and subway operations, size of the transport fleet, and so forth, aiding in the realistic simulation of public transit dynamics and its interaction with other transportation modes.

For the autonomous vehicle component of the simulation, the setup mirrored Shanghai’s One Card System’s framework, establishing electric vehicle autonomy at a 300-kilometer range and setting charging velocity at 30 kilometers per hour, based on current technology and equipment standards.

To streamline the computation process and boost efficiency, the simulation adopted the UTM Zone 51N projection, converting all geographic coordinates into this format to facilitate faster and more straightforward distance calculations [21].

#### Agent travel activity data support

For the simulation, communication data offers insights such as the timing, base station locations, and types of events. With over 16,000 base stations, the city’s coverage is mapped into Voronoi polygons, ensuring each area is closest to its designated base station, facilitating accurate movement tracking as users transition across coverage zones, with the user’s device reporting time and base station changes.

Mobile data from a typical April 2021 workday was analyzed to formulate preliminary patterns of activities and travel. This analysis deduced location, timing, activity categories, and transportation methods [22,23], categorizing activities into work, home, or other and identifying transportation modes as either vehicular, pedestrian, or cycling.

**Fig 3:**
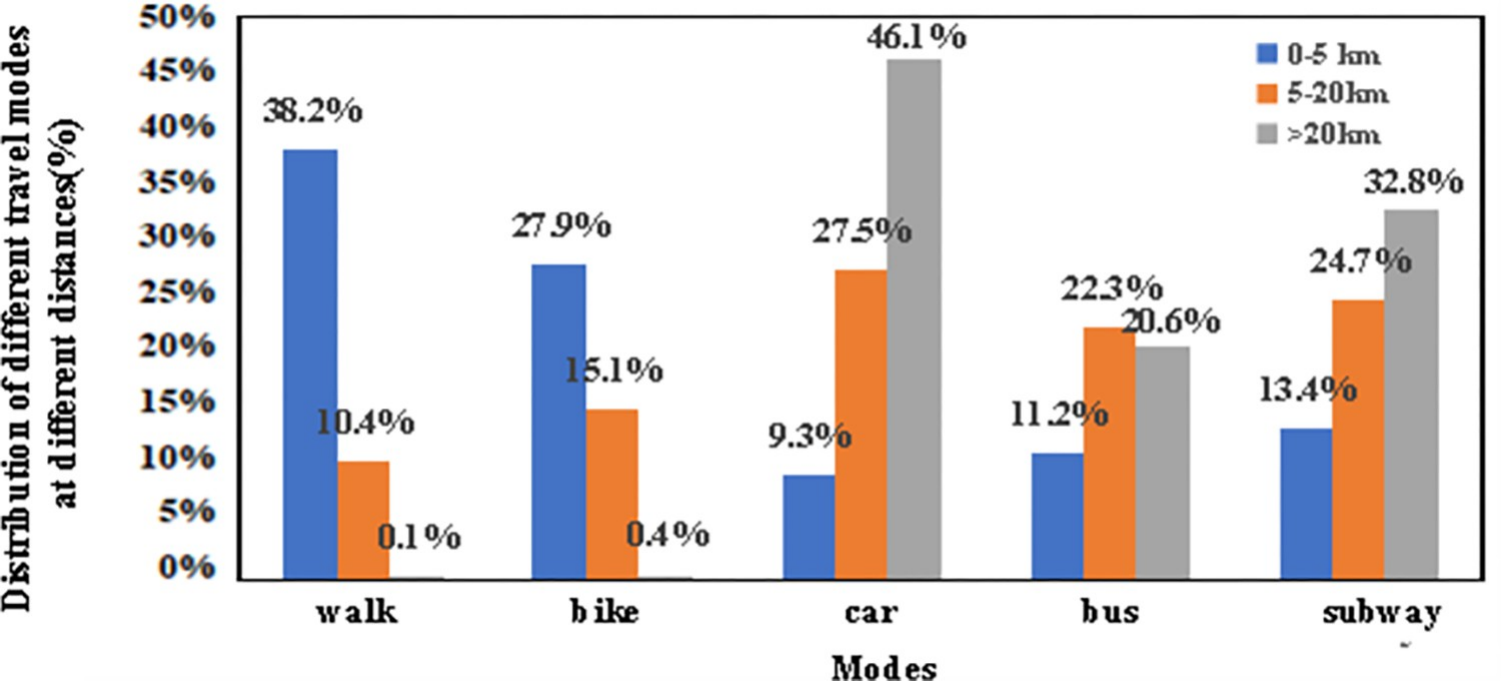
Distribution of different travel modes at different distances.

Modifications were applied in two key areas:

1. Activity Locations: The geographic points from the data, representing control points of polygons rather than precise activity locations, were assigned to matching activity-related facilities using Points of Interest (POI) data. This encompassed residential, workplace, educational, dining, shopping, and recreational facilities.
2. Transportation Modes: Given the generic categorization into buses, subways, and cars, a more detailed classification was needed. Utilizing the city’s sixth traffic survey, we distributed these modes across various travel distances, assigning transportation types based on distance and a calculated probability distribution.As shown in Fig 3.

The cellphone data represented about 2.6 million people, roughly 11% of the city’s population. To align with the simulation’s scope—especially considering the limited parking for autonomous electric vehicles (simulated at five spots per site, against a potential for ten)—the modeled population was scaled to represent 50% of city residents, adjusting activity sites and modes, and departure times randomly within predefined ranges for realism. Road usage and car availability were also scaled down to mirror actual conditions.

Due to computational constraints, the simulation, which utilized two Intel Xeon Gold 6226R CPUs and 300GB of RAM, did not encompass the entire population, focusing instead on a representative subset to achieve a balance between detailed simulation and resource availability.

#### Parameter configuration

Parameter Configuration In MATSim, the plans of each agent are evaluated after each iteration using a utility function. The utility function was developed by Harypar and Nagel (2005) and is roughly based on the road congestion Vickrey model described by Vickrey (1969) and Arnott (1993). The basic function is given by:

$$S_{plan}=\sum_{i=1}^{m}{(S}_{plan,i}+S_{travel,i})$$ (11)

This function encompasses the positive utility derived from activities and the negative utility arising from travel. The travel component considers factors such as travel time, travel cost, and travel distance. The travel cost between two activities can be expressed as follows:

$$S_{\mathrm{trav},q}=C_{\mathrm{mode}(q)}+\beta_{\mathrm{trav},\mathrm{mode}(q)}\cdot t_{\mathrm{trav},mode(q)}+\beta_{m}\cdot (\Delta m_{q}{+\gamma }_{d,mode(q)\cdot d_{\mathrm{trav},q}})$$ (12)

In this context, C_mode (q)_ represents a constant specific to a particular mode, β_trav, mode (q)_ is served as the direct marginal utility of travel time in that mode. t_trav, mode(q)_ denotes the travel time between two activities, Δm_q_ represents the monetary budget variation induced by ticket prices. β_m_ is the monetary budget variation induced by ticket prices. γ_d, mode (q)_ represents the monetary distance rate in a specific mode. d_trav, q_ stands for the travel distance between two activities. The utility function for autonomous electric vehicle services exhibits certain differences. The utility of travel in autonomous electric vehicle journeys is defined as:

$$S_{\mathrm{trav},q,ev}=C_{ev}+\beta_{m,ev}\cdot C_{t}{\cdot t+\beta_{t,ev}\cdot t+t}_{\mathrm{trav},mode(q)}+\beta_{d,accessoregresswalk}\cdot (d_{a}-d_{e})$$ (13)

Within this context, C_ev_ represents a constant. β_m,ev_ represents the marginal utility of spending an additional unit of funds on car travel. C_t_ denotes the monetary cost associated with rental time, where t represents the rental time, equivalent to the time spent inside the vehicle, β_t,ev_ expresses the marginal utility of spending an additional unit of time for car travel. The final component assesses the ingress and egress distance.

For the monetary cost parameters in the utility function, γ_d, mode (car)_ is set at 0.86 Chinese Yuan per kilometer, solely related to fuel consumption. The overall cost, including ownership, insurance, and maintenance, is 2.76 Yuan per kilometer. γ_d, mode (pt)_ is set at 0.24 Chinese Yuan per kilometer, Δm_qt_ is set as 2.74 Yuan Chinese Yuan. These values are derived through cost and travel distance regression from the Baidu Maps application programming interface (API). Additionally, C_t_ is set at 0.7 Chinese Yuan per minute, equivalent to the actual monetary cost of renting a car per minute.

#### Simulation scenario design

The provided tools and foundational scenario set the stage for simulating the dynamics of one-way electric autonomous vehicle (AV) sharing. This groundwork allows for the development of additional scenarios aimed at investigating how modifications on the supply side of AV sharing might influence consumer behavior, with the goal of refining shared vehicle services.

Given the shift towards electric vehicles (EVs), the AV ecosystem faces new limitations related to battery capacity and the efficiency of charging infrastructure. The initial scenario examines the impact of enhanced vehicle range and charging rates on the adoption of electric AV sharing. Within the established baseline, EVs are capable of 200 km with a charging velocity of 21 km/h. This study introduces two hypothetical scenarios to test changes: the first extends the EV range to 400 km while maintaining the charging speed, and the second maintains the 200 km range but increases the charging speed to 42 km/h.

EVs typically adhere to one of two charging approaches: the standard charging mode and the battery swap method. The latter involves swapping out batteries that are low on charge during times of minimal vehicle use, like during certain hours, thus eliminating the need for charging stations and reducing both initial and ongoing costs. This method also addresses the issue of EVs occupying charging bays longer than necessary or users failing to charge the vehicles after use. Nevertheless, the swap method raises questions about its ability to meet the daily operational demands of shared vehicles without the need for charging. Consequently, a "battery swap scenario" is conceptualized, maintaining a 200 km range but with no inherent charging capacity, to assess potential shifts in user travel patterns within this alternative charging framework.

## Results

### Dynamic travel model results

**Fig 4:**
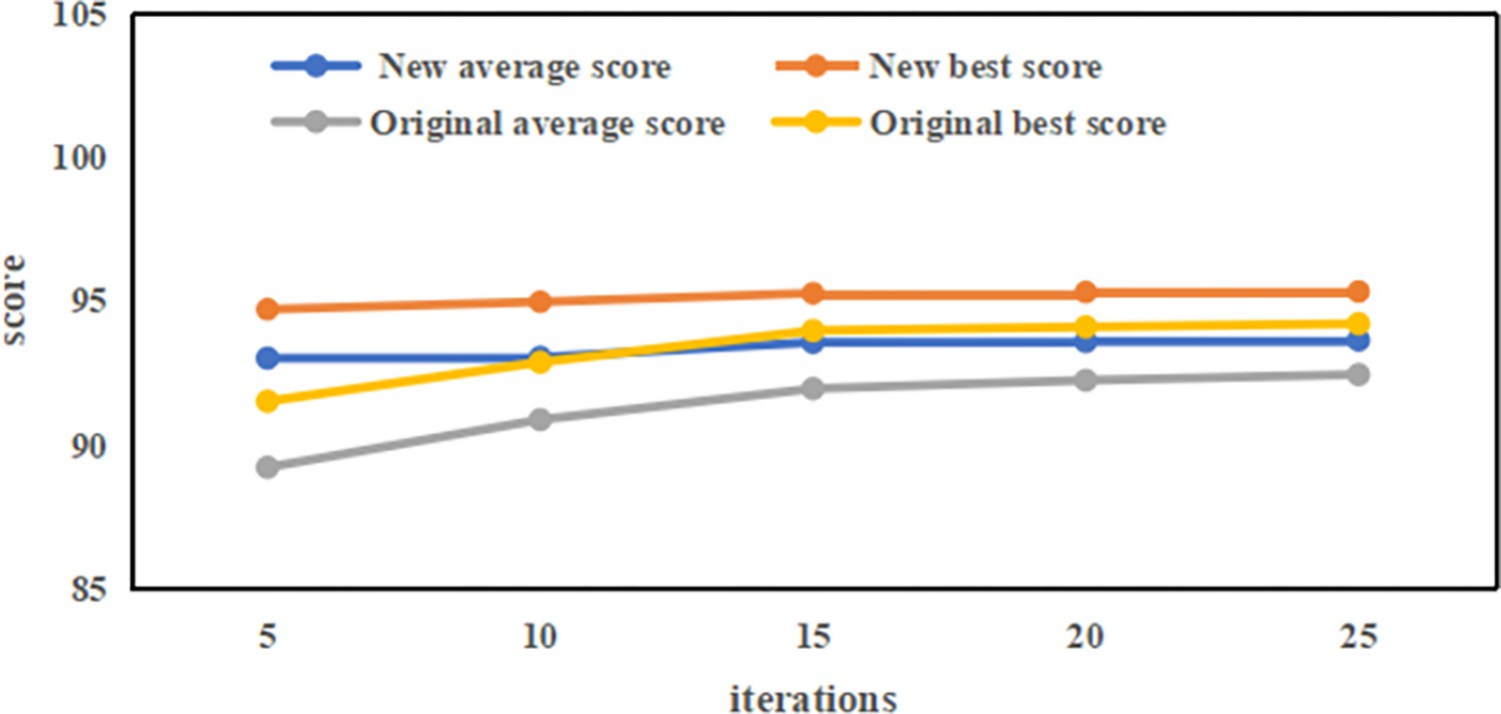
Comparison chart of scores between new and original methods.

The "original methods" refers to MATSim’s default settings where the number of plans generated by agents is either not set or fixed at five. The "new method" refers to our proposed approach of dynamically setting the number of plans generated by agents. As illustrated in the Fig 4, the enhanced technique achieves the top score more swiftly, showing an approximate 2% improvement over the traditional method. This boost in planning scores indicates a greater fulfillment of agents’ travel requirements and a decrease in trip cancellations caused by planning discrepancies. The diminished need for multiple iterations conserves computational resources and heightens the efficiency of the process.

### Autonomous driving model results

The results consist of two parts: (1) Validation of the Shanghai baseline scenario, including comparisons of mode split, traffic flow, and characteristics of autonomous vehicle travel. (2) Simulation results of the proposed scenarios.

#### Model validation

**Fig 5:**
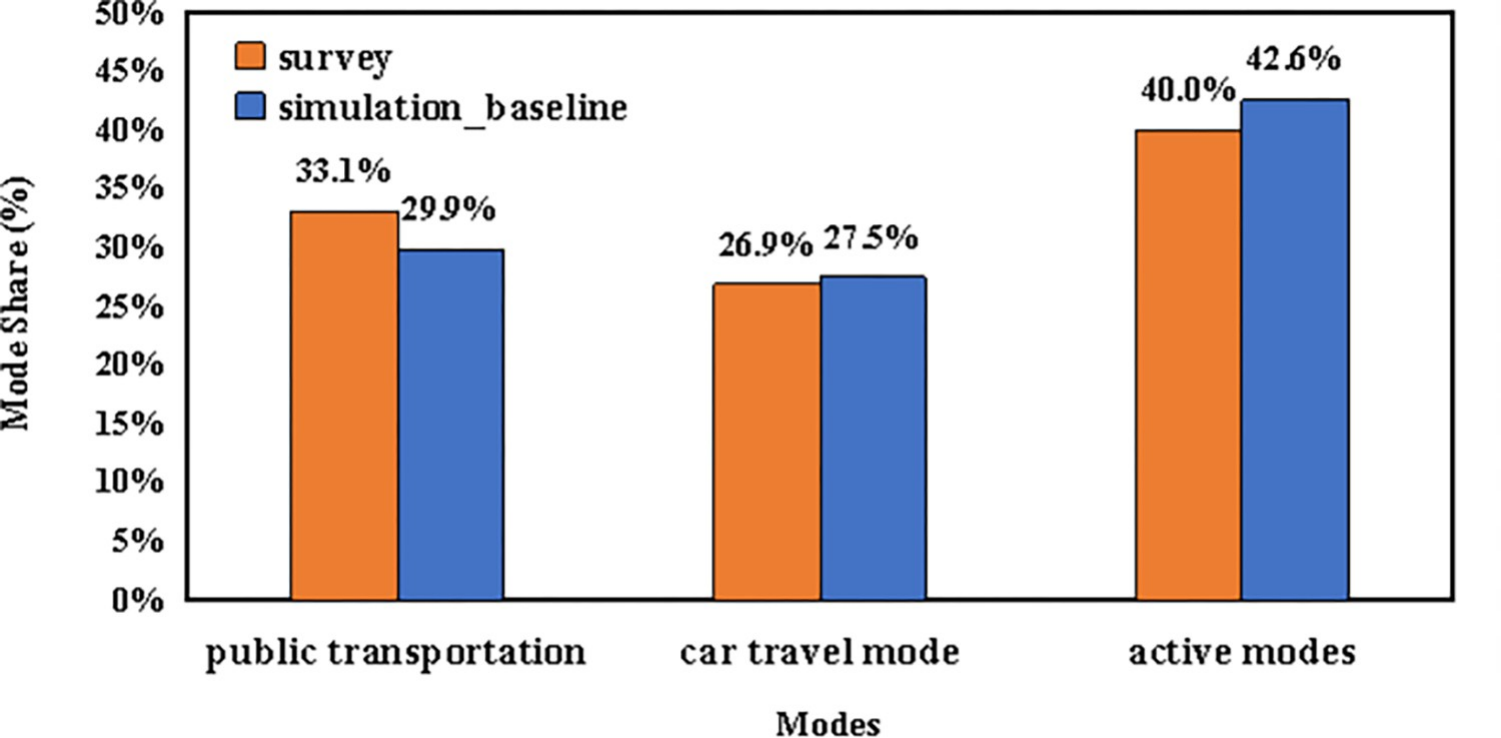
Illustrates the mode split between the simulated scenario and the travel diary data from Shanghai in 2020.

Fig 5 presents a comparison of transportation mode usage between the baseline scenario and findings from the sixth Shanghai Comprehensive Transportation Survey conducted in 2020, both relying on data at the trip level. The comparison reveals an underestimation in the use of public transit and active transportation methods such as biking and walking, while overestimating the use of private vehicles in the simulation results compared to the survey data. With ongoing enhancements and expansion in Shanghai’s public transit infrastructure, the usage of public transportation is on an upward trend. Furthermore, the data underpinning the demand originates from mobile phone tracking, which may not capture short journeys undertaken by active modes within a single base station’s coverage, thus potentially omitting them from the analysis.

**Fig 6:**
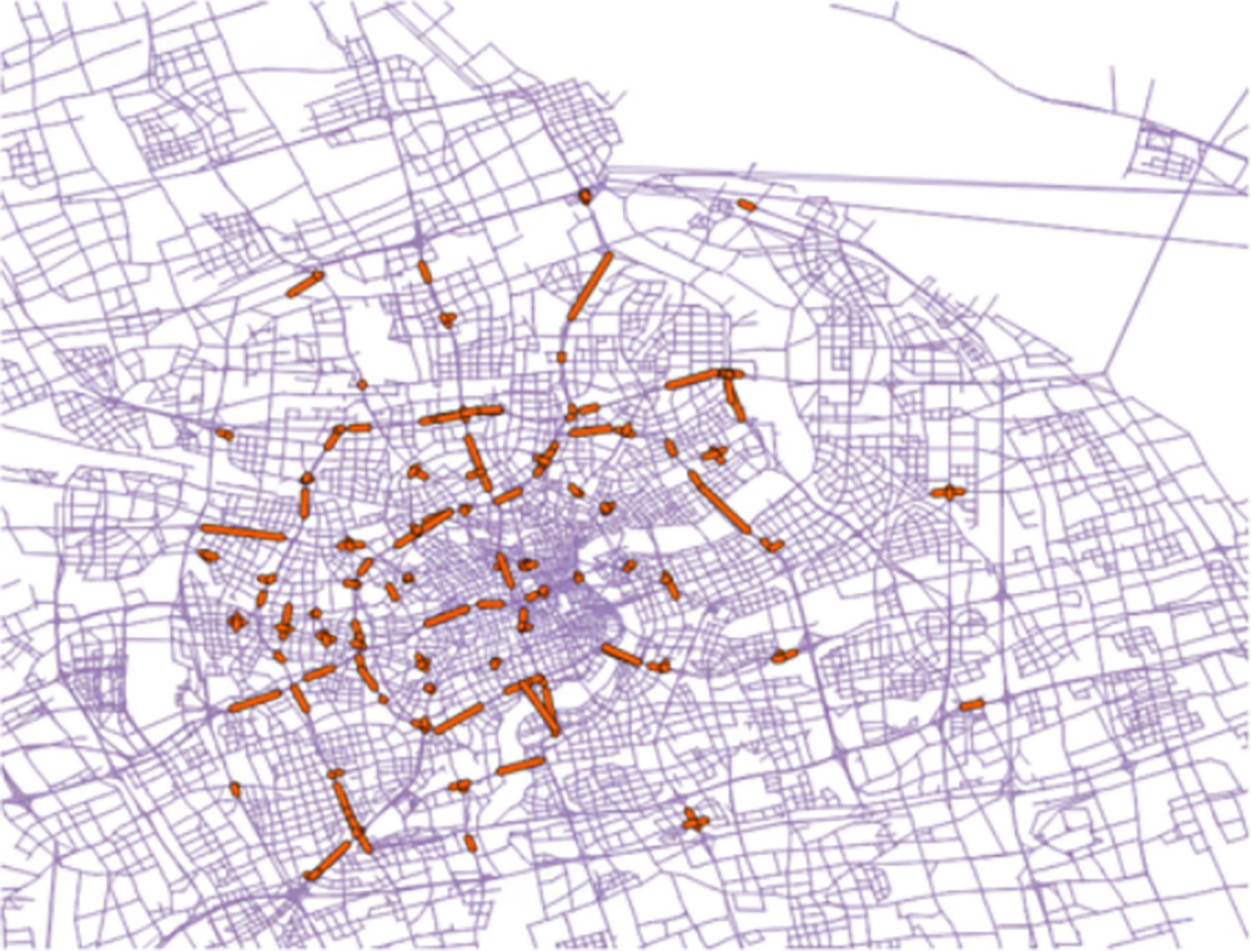
Depicts the distribution of counting stations in Shanghai.

Fig 6 depicts how counting stations are distributed throughout Shanghai, highlighting that the city hosts 344 such stations. Among these, 96 are located on elevated expressways, while the remaining 248 are positioned at ground level. The data collected from these stations primarily focus on the traffic flow of light vehicles observed during the morning peak hours, specifically between 8 and 9 a.m.

**Fig 7:**
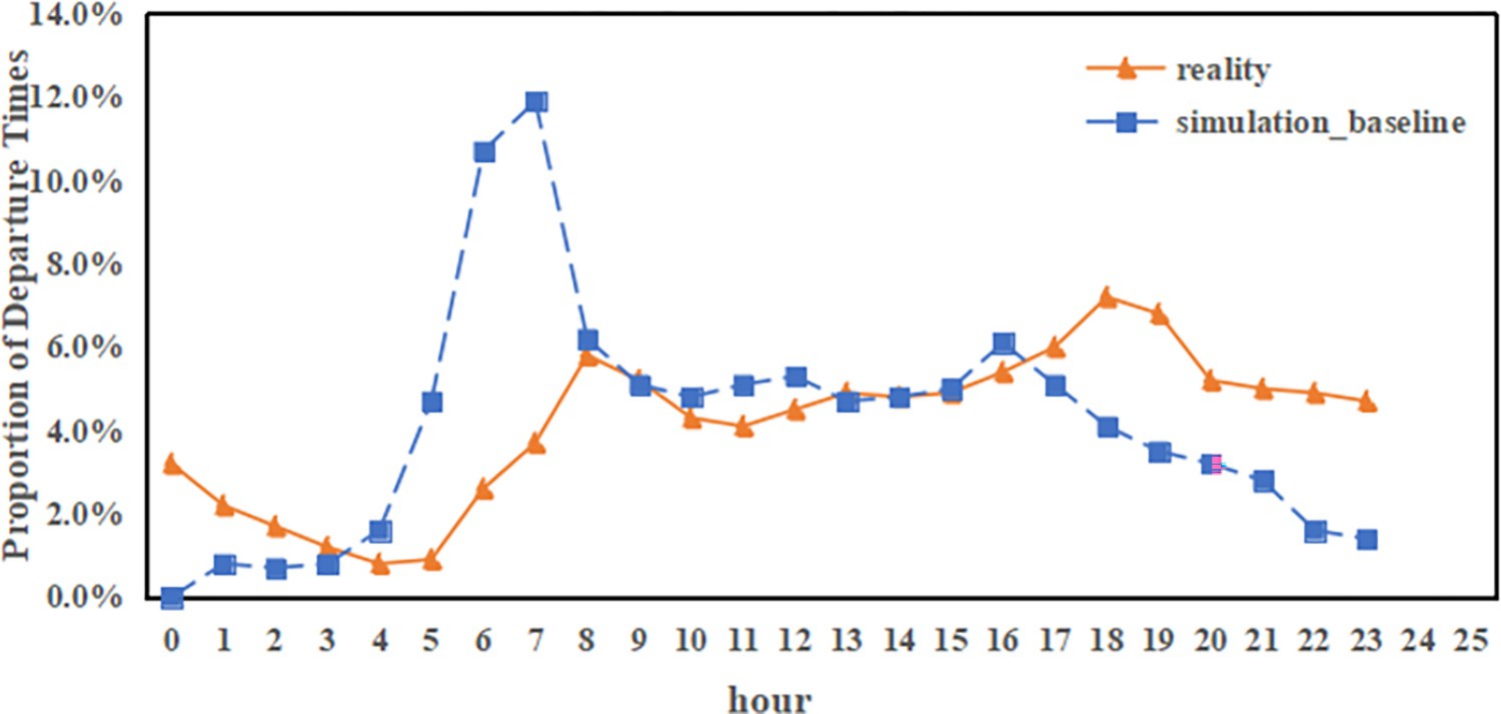
Comparison of the carsharing departure time in the simulation with reality.

The comparison between simulated traffic counts and actual observations, as illustrated in Fig 7, reveals that the discrepancies between these two data sets average at 18.6% for elevated expressways and 32.1% for ground-level roads. This indicates that the simulations for elevated expressways generally stay within a 20% margin of error, whereas simulations for ground-level roads exhibit greater variation from real-world counts. Referencing a validated MATSim model for ground-level roads developed by Jonas et al. [24] in Barcelona, which reported an average difference of 36.1%, and studies by Blaise et al. that found a typical prediction error exceeding 20% in traffic planning models, this simulation’s performance is comparatively effective for city-wide analyses. It offers valuable insights for quickly assessing the impact of new technologies and policy changes on urban traffic dynamics.

**Fig 8:**
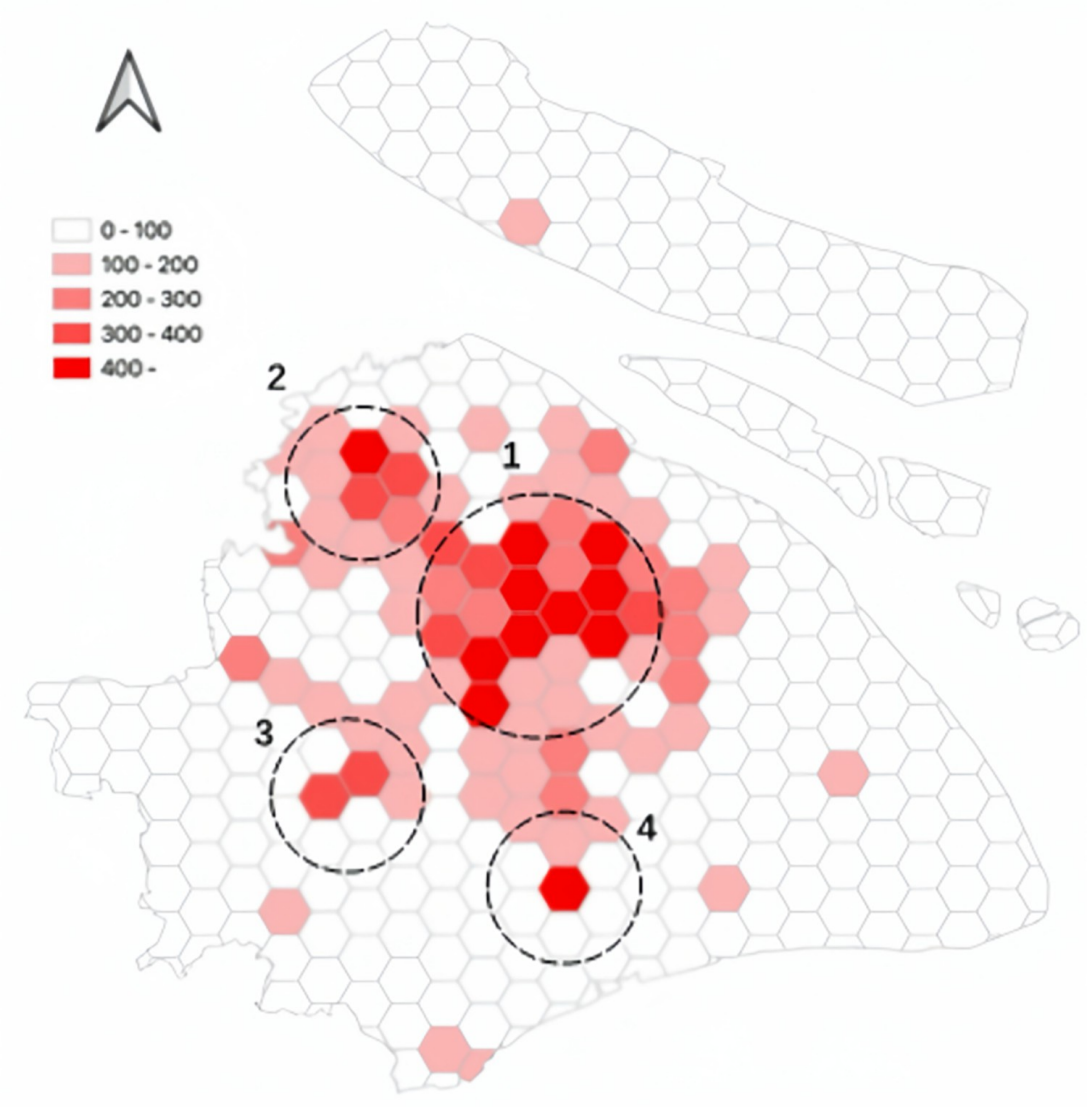
The spatial distribution of trips made by autonomous electric vehicles.

Fig 8 shows the spatial distribution of trips made by autonomous electric vehicles. In the hexagonal grid, simulated trips made by autonomous electric vehicles are depicted. Trips made by autonomous electric vehicles are primarily distributed in the following areas: 1- City Center, 2- Jiading District, 3- Songjiang District, and 4- Fengxian District.

**Fig 9:**
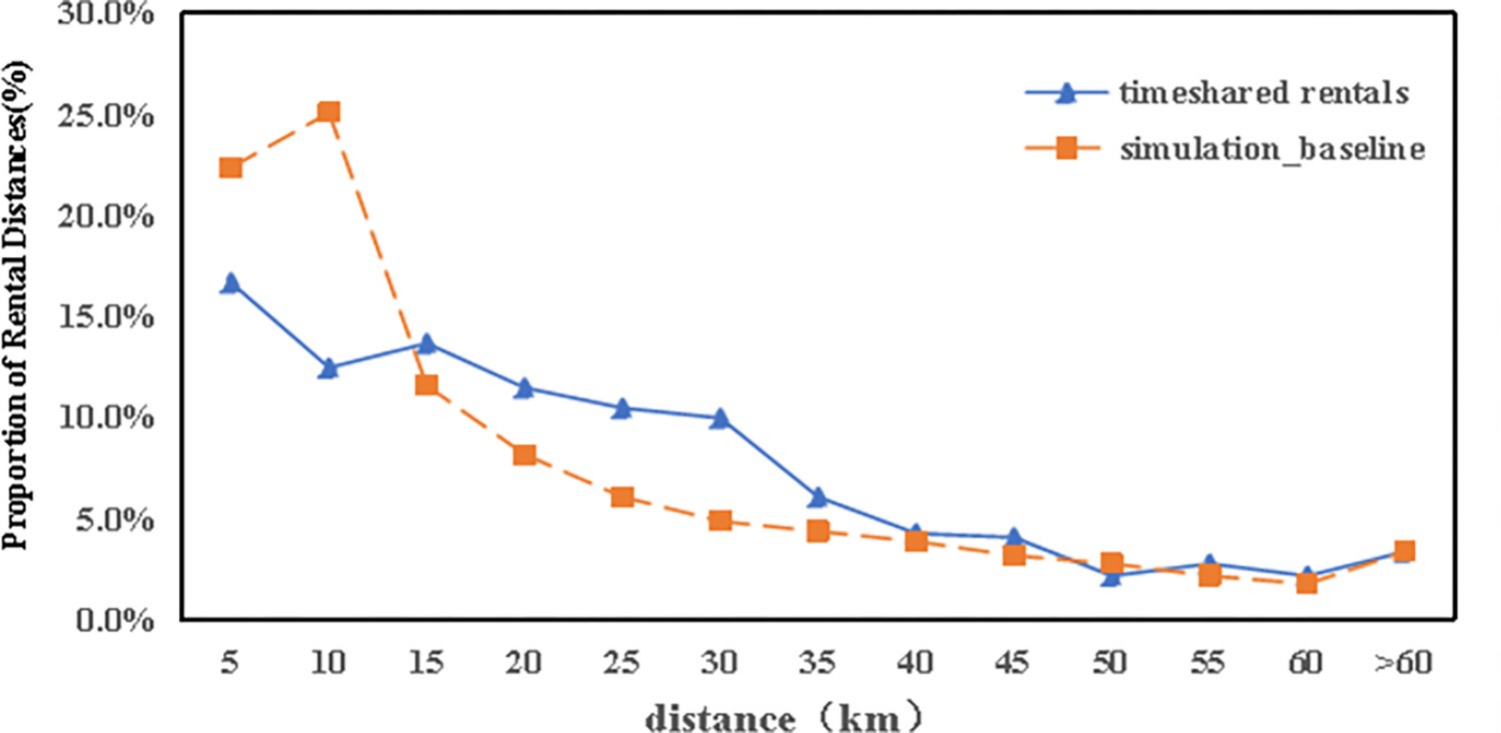
Comparison of the rental distance in the simulation with timeshare rentals.

Fig 9 demonstrates that the usage rate of autonomous electric vehicles declines with longer travel distances. Comparatively, data from the city’s existing car-sharing service shows an average journey length of 15.1 kilometers, whereas the simulation indicates an average of 18.7 kilometers. This difference is attributed to the utility function embedded within the car-sharing simulation model. Considering the pricing mechanism of the actual car-sharing service, which calculates fees based on the duration and distance of rentals, users are naturally inclined to limit both the length and time of their journeys to reduce costs.

### Simulation scenario results

#### Power usage of autonomous electric vehicles

**Fig 10:**
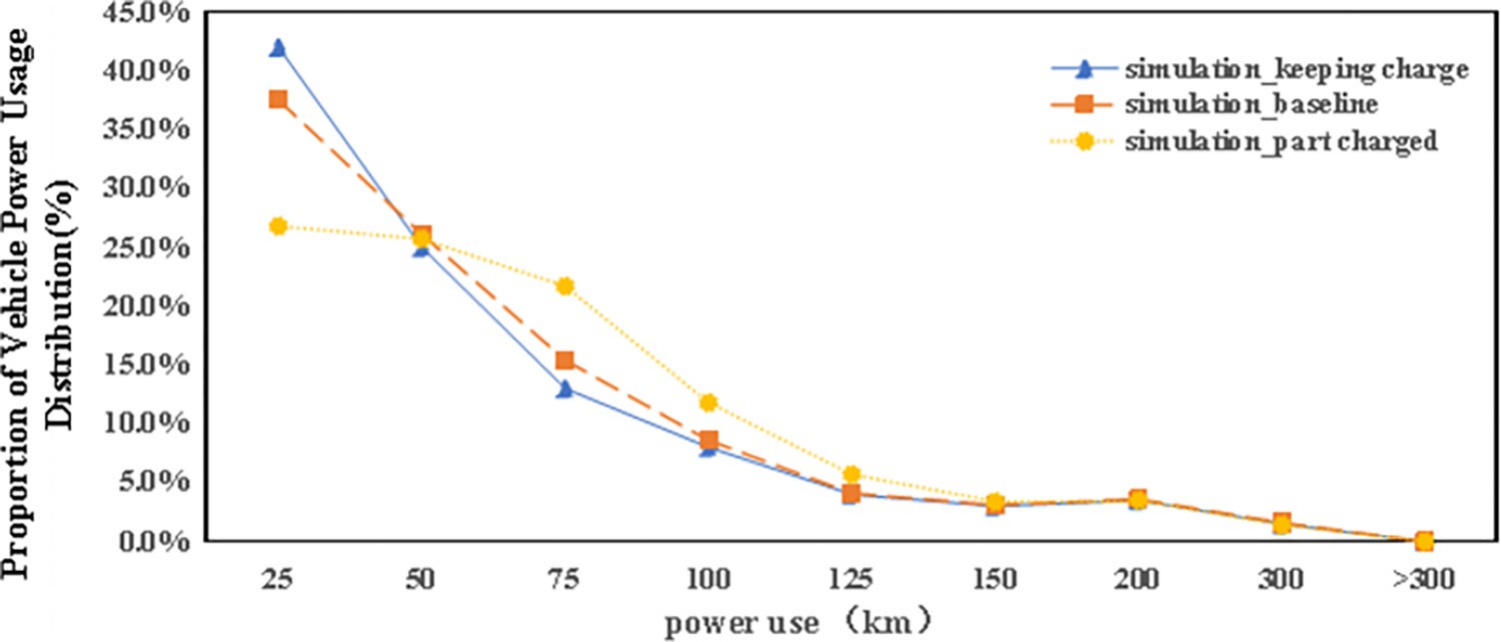
The distribution of autonomous vehicle electric power use in baseline scenario.

Prior to delving into the outcomes of the specifically designed scenarios, an overview of electric power consumption by carsharing vehicles in the baseline scenario is provided. As depicted in Fig 10, a notable trend is observed where the percentage of electric vehicles (EVs) decreases as their power consumption increases, with nearly 40% of vehicles utilizing less than 25km of power in total. Despite the availability of charging support, the majority of vehicles are operated for distances under 150km, with only a minority exceeding this total power consumption.

Carsharing stations feature designated parking spots for carsharing vehicles. When these spots are all occupied, additional returned vehicles may resort to using adjacent legal parking areas without charging amenities, leading to a categorization of EVs into “keeping charged” vehicles (those that are always returned to charging stations) and “part charged” vehicles (those returned to non-charging parking spaces). Interestingly, Fig 10 illustrates that the power usage among part charged EVs exceeds that of their keeping charged counterparts, particularly for distances ranging between 50-100km. This observation is somewhat unexpected since vehicles that are not fully charged are presumed to be used less frequently. However, analysis reveals that part charged vehicles are utilized more often, at a rate of 3.2 times per day, compared to 2.6 times per day for keeping charged vehicles. This higher usage rate can be attributed to the strategic location of drop-off stations prone to overflow, which are often among the most frequently used. Thus, part charged vehicles, found primarily in areas with high carsharing demand, are likely to be booked again swiftly, explaining their elevated usage rates compared to fully charged vehicles.

#### Results of the extended coverage and accelerated charging scheme

The study explored two distinct scenarios: (1) The Extended Range (ER) charging scenario and (2) the Accelerated Charging (AC) scenario, to assess the impact of enhanced vehicle range and faster charging rates on the usage of shared autonomous vehicles. This included aspects such as the volume of shared trips, their spatial and temporal patterns, travel distances, and energy consumption. Analysis of these scenarios revealed that extending the vehicle’s range (ER scenario) led to a modest increase in shared trips by 1.2%, whereas improving the charging speed (AC scenario) had negligible effect on the overall demand for shared autonomous vehicle services. This suggests that modifications in both vehicle range and charging speed have limited influence on user demand for these services.

**Fig 11:**
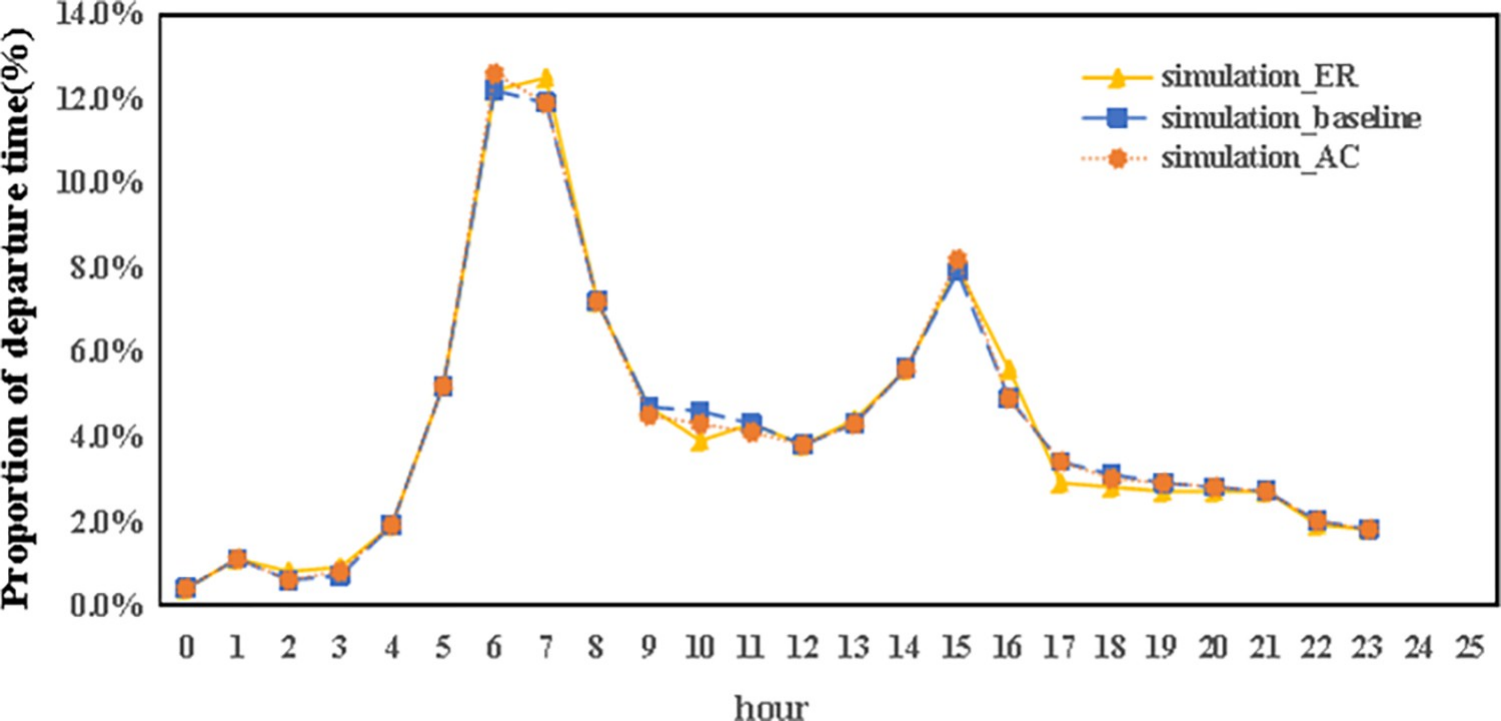
The departure time of autonomous vehicles in the ER and AC scenarios.

**Fig 12:**
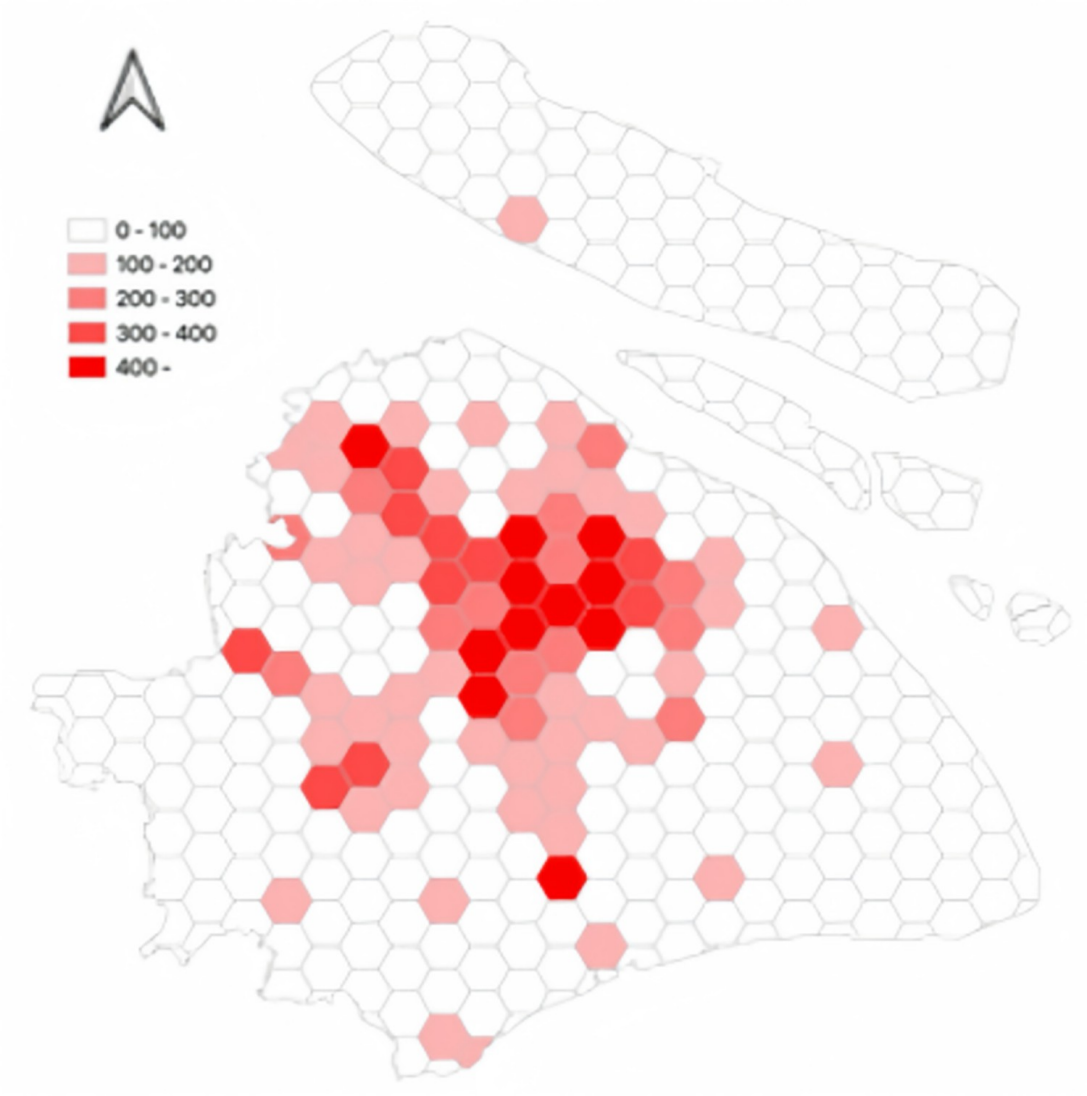
The spatial distribution of autonomous vehicles in the ER scenarios.

**Fig 13:**
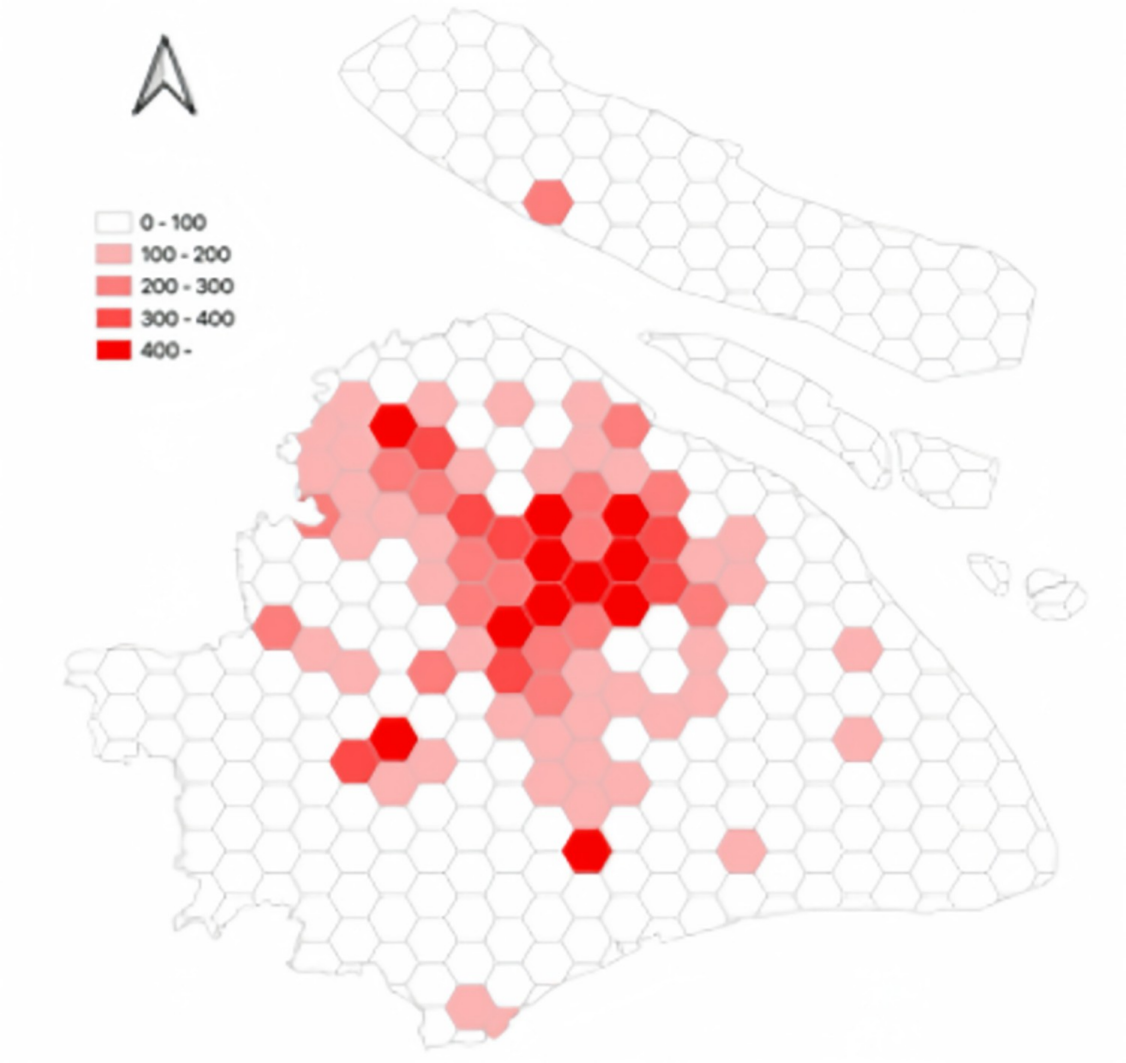
The spatial distribution of autonomous vehicles in the AC scenarios.

**Fig 14:**
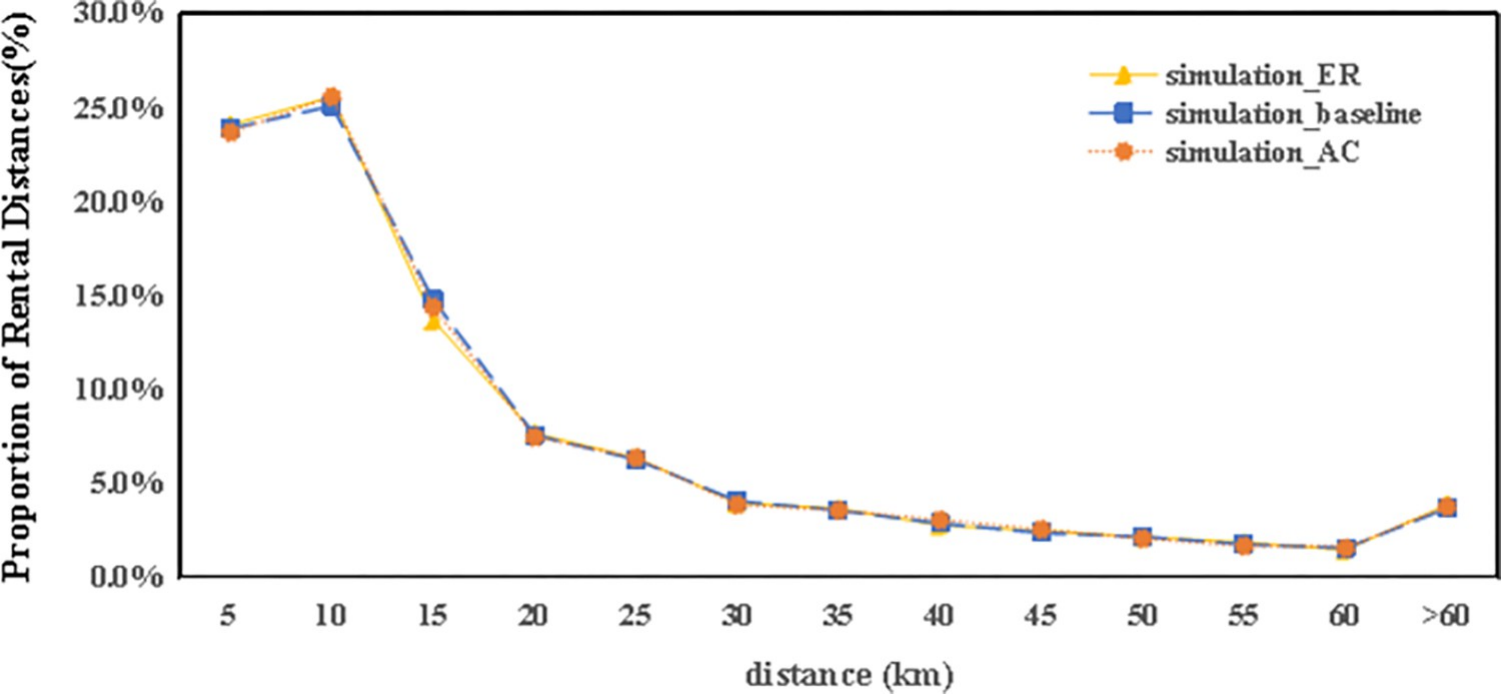
The distance traveled by autonomous vehicles in the ER and AC scenarios.

**Fig 15:**
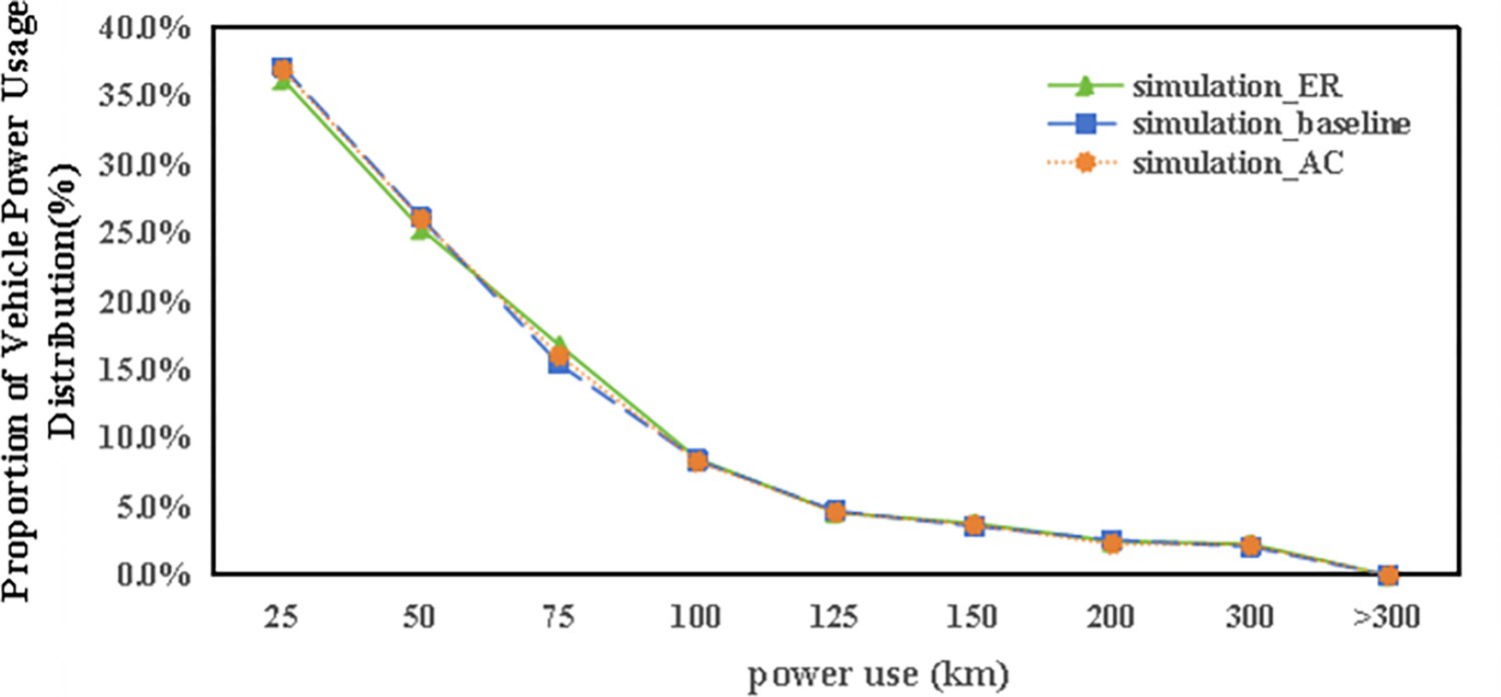
The distribution of autonomous vehicle electric power use in ER and AC scenario.

Closer scrutiny of the data, including the spatial layout of autonomous vehicle trips (referenced in Fig 11), their time distribution (Figs 12 and 13), and the range of travel distances (Fig 14), showed that travel patterns remained largely consistent with those observed in the baseline scenario. Additionally, the distribution of energy use (Fig 15) demonstrated a minor uptick in the fraction of vehicles consuming over 200 km of power following the range extension, alongside a decrease in the usage of vehicles drawing less than 50 km of power.

These findings imply that the existing specifications for electric vehicle range and charging capabilities are sufficient to cater to the daily commuting needs within Shanghai. Therefore, enhancing the range and charging speed of these vehicles is unlikely to result in significant shifts or advantages for the shared autonomous vehicle sector.

#### Battery replacement scheme results

**Fig 16:**
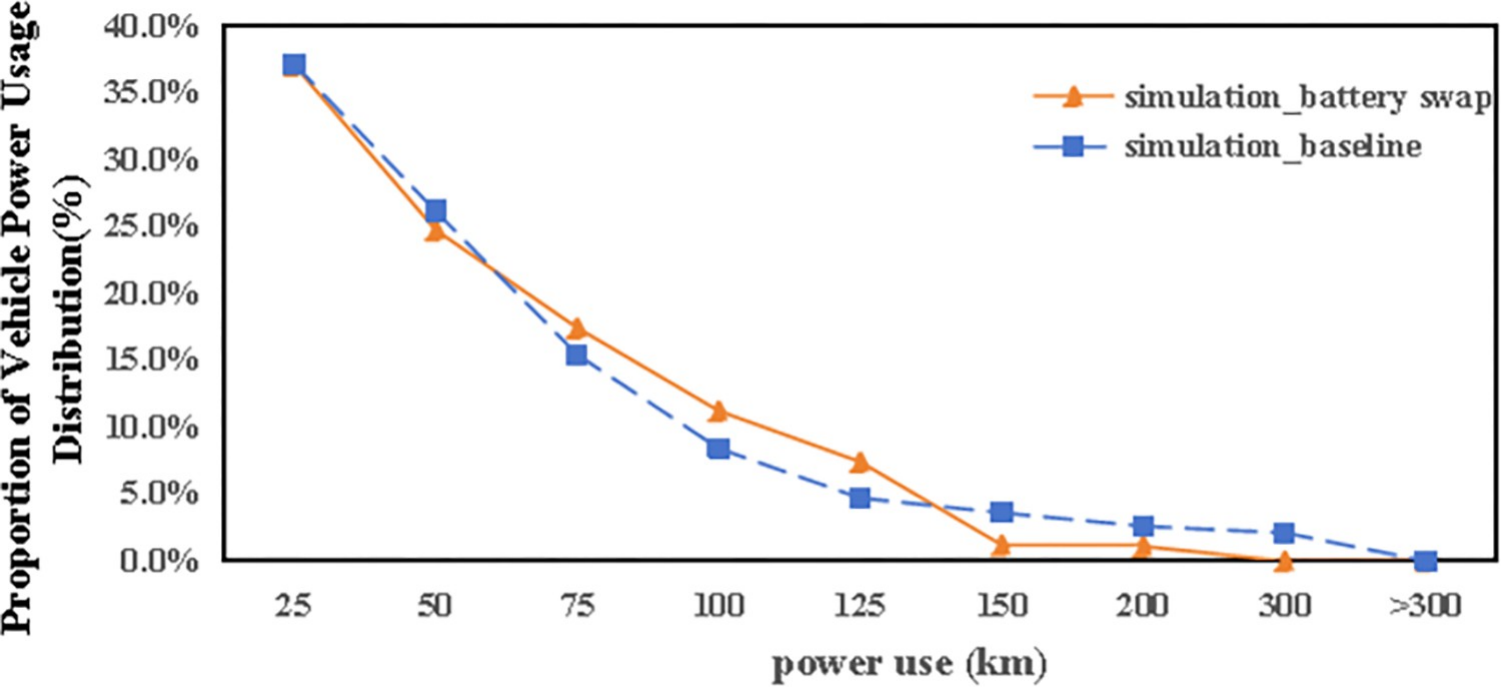
The distribution of autonomous vehicle electric power use in battery swap scenario.

Fig 16 presents how energy consumption patterns among autonomous electric vehicles adapt within a battery swapping framework, characterized by the elimination of traditional charging processes (charging speed is assumed to be 0 km/h). The shift towards battery swapping has led to a slight reduction, about 2.5%, in the overall number of vehicle trips. Analysis of the energy consumption data under this new scenario reveals several key points:

1. With the non-reliance on charging stations, there’s virtually no instance where vehicles expend energy beyond their maximum range capacity of 200 kilometers, indicating effective energy management.
2. The percentage of vehicles utilizing energy within the 50–125 kilometers bracket drops significantly, underscoring the reduced need for mid-range energy consumption in the absence of conventional charging stations.
3. Conversely, a noticeable increase in vehicles using energy in the 25–125 kilometers range suggests an enhanced usage rate for vehicles within this energy consumption window, compared to the standard scenario. Meanwhile, the share of vehicles operating within the lower energy bracket (25–50 kilometers) diminishes.
4. The battery swapping approach also reflects a decline in the usage of vehicles with minimal remaining energy (those traveling less than 25 kilometers), indicating a preference or necessity for vehicles with sufficient charge to meet the journey requirements of users.

These insights imply that, within the battery swapping model, there’s a strategic utilization of vehicle energy, with preferences leaning towards vehicles that can cover more distance without the need for immediate recharging. This shift could potentially streamline operations and ensure that vehicles are optimally charged and ready for use, meeting the travel demands of users more efficiently.

**Fig 17:**
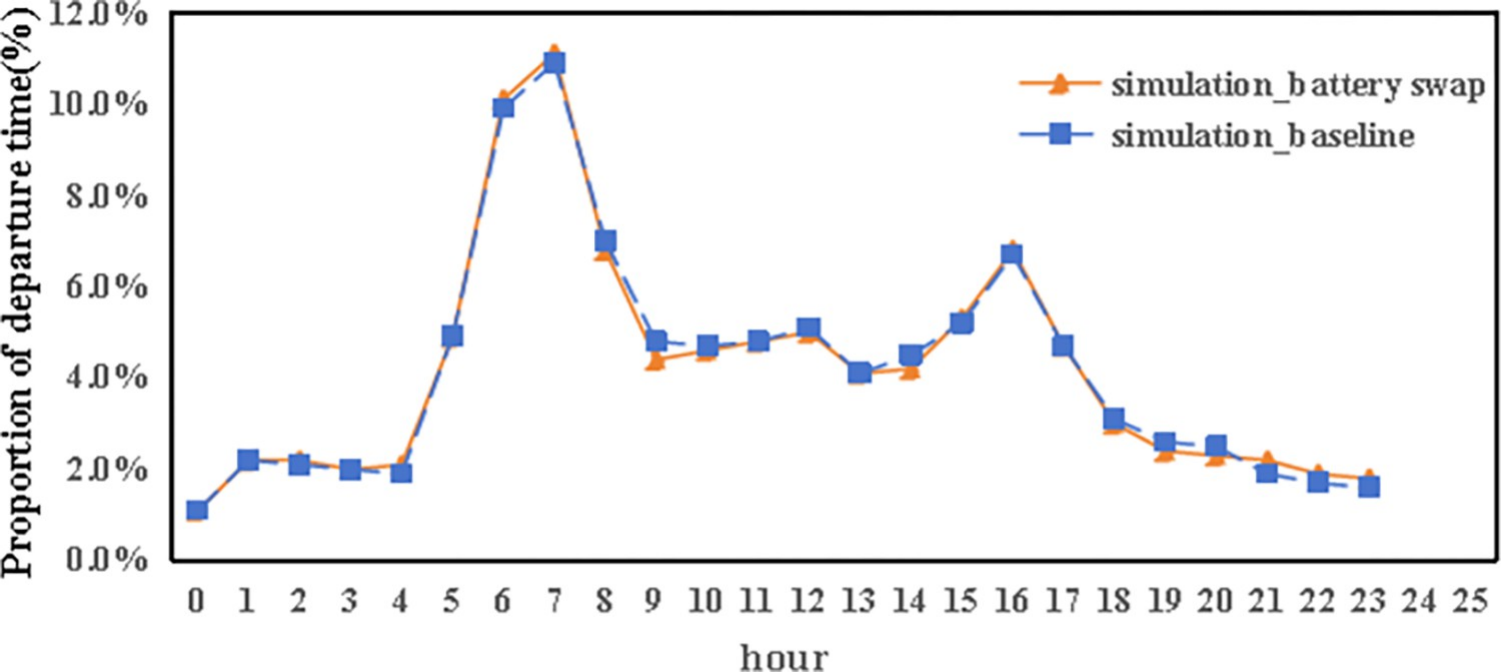
The departure time of autonomous vehicles in the battery swapping scenario.

**Fig 18:**
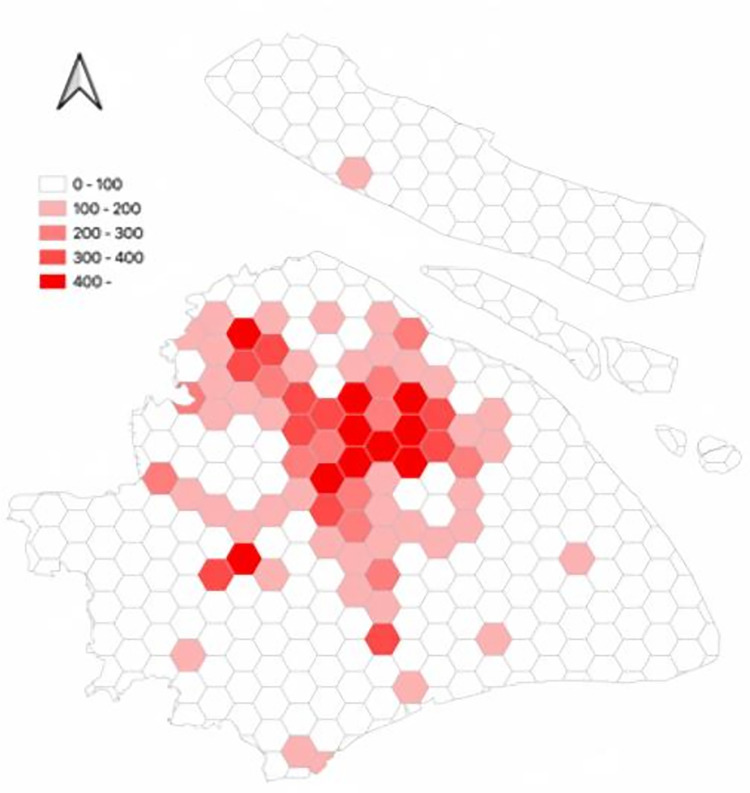
The spatial distribution of autonomous vehicles in the battery swapping scenario.

**Fig 19:**
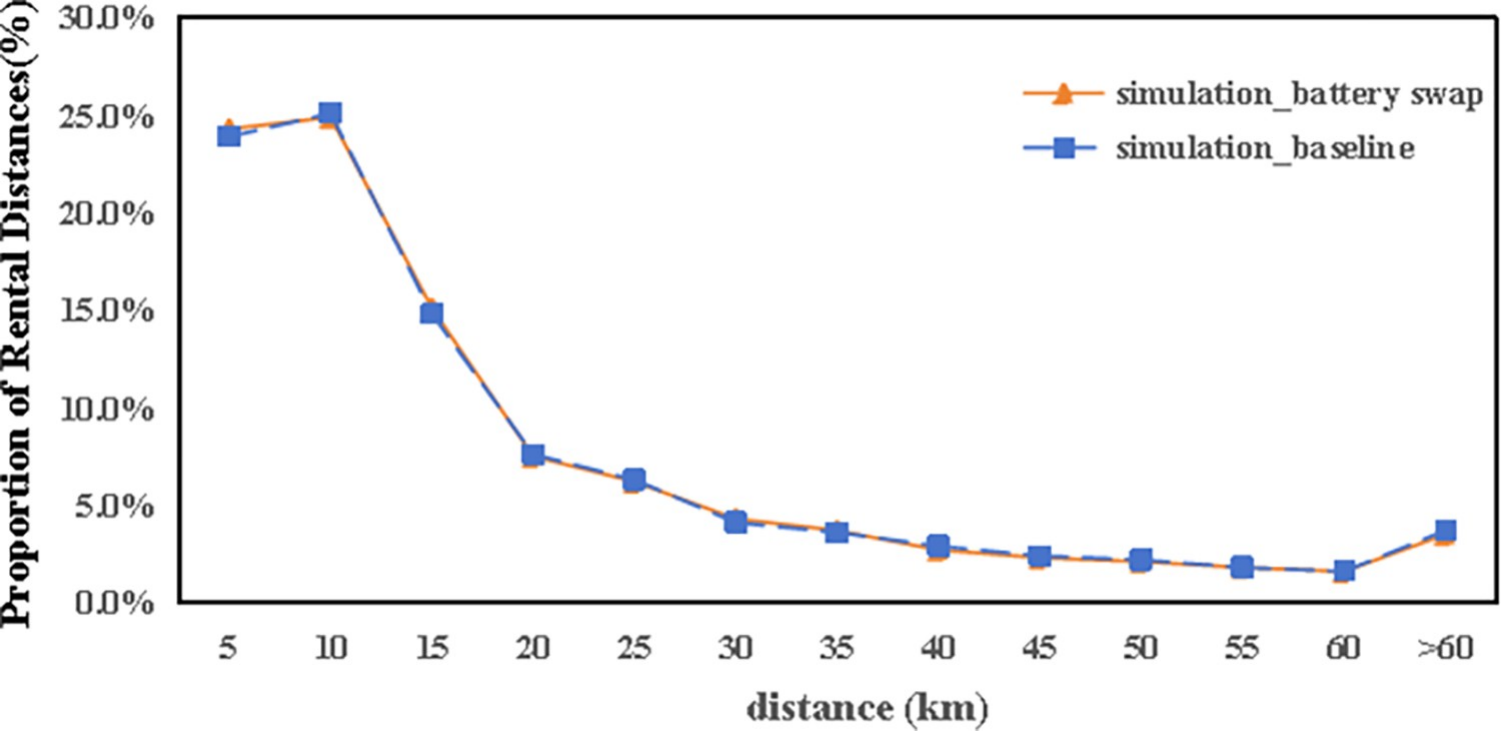
The rental distance in battery swap scenario.

Furthermore, an analysis of the spatiotemporal patterns (illustrated in Figs 17 and 18) and the distribution of travel distances (shown in Fig 19) for shared autonomous vehicles operating under a battery swapping scenario has been conducted. The investigation reveals that the fundamental travel behaviors associated with shared vehicle usage are consistent, even with the shift in energy replenishment methods. This transition from conventional charging stations to a battery swapping mechanism does not significantly impact the overall volume of vehicle trips. The findings suggest that adopting battery swapping as an alternative power supply strategy effectively supports the existing demand for shared vehicle services, adequately catering to daily transportation needs within Shanghai. This adaptability underscores the potential of battery swapping to maintain operational continuity and user service levels in urban shared mobility systems.

From the results and analysis above, we can conclude the following: First, implementing dynamic travel strategies on the MATSim platform optimizes vehicle dispatching and user demand matching, aiding urban planners and traffic managers in developing more efficient traffic flow strategies. Second, although enhancing electric vehicle range and charging speed has limited impact on their usage, adopting a battery swapping system is a viable alternative. Urban planners should consider investing in this infrastructure. Lastly, the study finds that existing vehicle specifications can meet daily urban travel needs. Therefore, substantial investments in extending range or increasing charging speed are unnecessary. Instead, resources should be better allocated to optimize current infrastructure and explore battery swapping methods. These discussions provide a strategic foundation for urban traffic managers, policymakers, and researchers to effectively integrate the shared autonomous electric vehicles model into urban transportation networks, ultimately achieving smarter and more efficient cities.

## Conclusion

This study contributes to the field of intelligent transportation systems by introducing an advanced approach for dynamic travel planning. Utilizing a novel method that dynamically adjusts travel plan generation, this research prioritizes high-scoring plans and minimizes the exclusion of potentially optimal solutions. This approach is instrumental in reducing traffic congestion, allowing for adjustments to travel plans in real-time based on actual conditions and individual preferences observed during simulations. Such detailed planning enhances traffic flow management, reduces congestion, and increases overall system efficiency, providing robust support for developing intelligent transportation strategies.

In addition, we developed a simulation module for autonomous electric vehicles tailored to address the specific challenges posed by charging infrastructure limitations. This module, integrated into the MATSim open-source simulation platform, facilitates virtual charging at designated stations, enabling users to verify if vehicles have sufficient power for planned journeys. Validated through a Shanghai-based scenario, this module supports realistic simulations of transportation network operations using actual traffic flow data.

The simulation leverages benchmark data and simulates transportation facilities to explore different scenarios, including extending vehicle range and modifying charging rates. Findings from these scenarios indicate that:

1. The simulation results closely align with real-world conditions, underscoring the model’s validity.
2. Adjustments to the range and charging speed of autonomous electric vehicles have minimal impact on usage patterns, confirming that current vehicle specifications meet Shanghai’s daily transportation needs.
3. Implementing a battery swapping system slightly reduces vehicle usage by 2.5% but does not significantly alter travel behaviors, suggesting that alternative charging strategies do not heavily influence current vehicle demand.

However, discrepancies between simulated and actual data highlight limitations within the current model, necessitating further research to enhance its reliability and applicability. Future work will focus on refining the model by optimizing parameters and incorporating additional factors such as varied driving conditions and environmental impacts, aiming to broaden the model’s utility and depth.
